# Association of overt hypothyroidism with risks of cognitive impairment: a meta-analysis and systematic review

**DOI:** 10.3389/fendo.2025.1643589

**Published:** 2025-10-01

**Authors:** Jinxin Zhu, Jialu Xu, Zhaoqing Li, Jia Liu

**Affiliations:** Department of Thyroid Surgery, General Surgery Center, The First Hospital of Jilin University, Changchun, Jilin, China

**Keywords:** hypothyroidism, cognitive impairment, Alzheimer’s disease, dementia, thyroid

## Abstract

**Background:**

Studies examining the relationship between overt hypothyroidism (oHT) and the risk of cognitive impairment (CI) have yielded mixed results. This study aimed to evaluate the association between oHT and the risk of CI.

**Methods:**

We systematically searched relevant studies published up to March 2025. Data were extracted independently by two investigators. Overall odds ratios (ORs) and their 95% confidence intervals (CIs) were calculated using a random-effects model. The Newcastle-Ottawa Scale (NOS) was used to assess the quality of cohort and case-control studies, while the Agency for Healthcare Research and Quality (AHRQ) scale was used for cross-sectional studies. Results were reported following PRISMA guidelines.

**Results:**

A total of 11 studies involving 1,190,059 participants were included in the systematic review. Meta-analysis revealed that oHT was associated with an increased risk of CI (OR = 1.18, 95%CI=1.04–1.34). When CI was categorized into mild cognitive impairment (MCI) and severe cognitive impairment (Alzheimer’s disease (AD) or all-cause dementia), oHT was associated with an increased risk of MCI (OR = 1.24, 95%CI=1.13–1.36) but not with AD (OR = 1.03, 95%CI=0.77–1.38) or all-cause dementia (OR = 1.20, 95%CI=0.94–1.53). Subgroup analysis based on diagnostic methods for oHT showed that oHT diagnosed solely by TSH levels was associated with a reduced risk of CI (OR = 0.87, 95%CI=0.79–0.95).

**Conclusion:**

Available evidence suggests an association between oHT and an increased risk of cognitive impairment, particularly MCI. However, given the observational nature and significant heterogeneity of this study, the strength of this association still requires high-quality prospective studies for final confirmation and precise quantification.

**Systematic review registration:**

https://www.crd.york.ac.uk/prospero/, identifier CRD420251012792.

## 1 Introduction

Cognitive impairment (CI) refers to a significant decline or dysfunction in memory, learning, attention, language, executive function, visuospatial ability, and other cognitive domains (1, 2). It can result from various factors, including neurodegenerative diseases [e.g., AD (3, 4), Parkinson’s disease (5, 6)], cerebrovascular diseases (7, 8) [e.g., stroke (9)], traumatic brain injury, infections, metabolic abnormalities (10, 11), drug side effects, and psychiatric disorders. The severity of CI ranges from mild cognitive impairment (MCI), which may only affect daily living efficiency, to severe dementia, which can lead to a complete loss of independent living ability. According to the World Health Organization (WHO), approximately 50 million people worldwide live with dementia, and this number is expected to rise to 152 million by 2050 (12). CI not only significantly impacts patients’ quality of life but also imposes substantial economic and caregiving burdens on families and society. Therefore, identifying modifiable risk factors and potentially reversible causes is crucial for delaying disease progression and improving patients’ quality of life.

Thyroid dysfunction is considered a potentially reversible cause of CI (13, 14). Guidelines from the American Psychiatric Association (APA), European Association of Neurology (EAN), World Health Organization (WHO), and National Institute for Health and Clinical Excellence (NICE) recommend thyroid function testing during CI evaluation. While the association between hypothyroidism and CI has been extensively studied (15, 16), whether hypothyroidism is an independent risk factor for CI remains controversial. Some studies suggest that hypothyroidism increases the risk of CI (14, 17), while others report no significant association (18, 19).

Previous meta-analyses have primarily focused on the association between subclinical hypothyroidism (sHT) and CI, with limited analysis of overt hypothyroidism (oHT) (20–22). There are significant differences in the pathophysiological mechanisms and clinical severity between oHT and sHT. Patients with oHT exhibit markedly insufficient thyroid hormone levels, which have a more pronounced impact on systemic metabolism and nervous system function (23). Therefore, this meta-analysis aimed to, as a preliminary effort, separately analyze the association between oHT and varying degrees of CI, which may have important clinical implications for preventing or delaying the progression of cognitive impairment.

## 2 Materials and methods underscore the importance of early diagnosis and treatment

### 2.1 Standard protocol approvals, registrations, and guidelines

This study adhered to the Preferred Reporting Items for Systematic Reviews and Meta-Analyses (PRISMA) guidelines and was registered prospectively with the International Prospective Register of Systematic Reviews (PROSPERO) (CRD420251012792).

### 2.2 Search strategy

We systematically searched PubMed, Web of Science, Scopus, Cochrane Library, and EMBASE for relevant studies published up to March 14, 2025. The complete search strategy is provided in the **Supplementary Table S1**. Additionally, references from included studies and existing systematic reviews were manually searched to identify additional relevant articles. No filters were applied to limit language, study type, country, or publication year.

### 2.3 Study selection and data extraction

Two independent investigators screened the literature, and any discrepancies in study inclusion were resolved through discussion or consultation with a third reviewer. After removing duplicates, studies were excluded if their titles or abstracts were irrelevant. Full texts were reviewed for eligible studies or when relevance could not be determined from the abstract alone.

Inclusion Criteria:

1. Cohort, case-control, or cross-sectional studies examining the association between hypothyroidism and CI.
2. Studies providing relative risk (RR), hazard ratio (HR), odds ratio (OR), or raw data to calculate these metrics, along with 95% confidence intervals (CIs).
3. Studies involving adults (age ≥ 18 years).
4. Studies reporting diagnostic criteria for oHT.
5. Studies published within the last 20 years (2005–2025).

Exclusion criteria:

1. Reviews, case reports, animal studies, letters, conference abstracts, or guidelines.
2. Studies lacking complete data or full-text availability.
3. Studies focusing solely on subclinical hypothyroidism (sHT).
4. Studies involving baseline conditions that may cause secondary hypothyroidism (e.g., pituitary or hypothalamic diseases).

Outcome definitions:

Cognitive Impairment: A broad term encompassing any clinically diagnosed decline in cognitive domains (memory, attention, executive function, etc.), assessed by standardized tools (e.g., MMSE, CDR). Mild Cognitive Impairment: Defined according to Petersen criteria or equivalent, characterized by subjective memory complaints with objective cognitive deficits not interfering with daily activities. Alzheimer’s Disease: Diagnosed based on NIA-AA criteria or ICD-10 codes. All-cause Dementia: Diagnosed using DSM-IV/ICD-10 criteria, regardless of etiology.

Extracted information included study year, author, country, study type, total sample size, follow-up duration, population age (median or mean), proportion of females, diagnostic criteria for oHT and CI, type of CI, and adjustment factors. In addition, we will employ specific formulas to convert HR values from cohort studies into OR values (24, 25), ensuring compatibility for subsequent pooled effect size analysis. When extracting OR values from the literature, both adjusted OR values (with the maximum number of covariates) and unadjusted OR values were comprehensively recorded.

### 2.4 Quality assessment

Two investigators independently assessed the quality of cohort and case-control studies using the NOS (26), which evaluates study population selection, group comparability, and exposure/outcome ascertainment. Studies were categorized as low (0–3 points), medium (4–6 points), or high quality (7–9 points). Cross-sectional studies were evaluated using the AHRQ checklist (27), which consists of 11 items scored as “yes,” “no,” or “unclear.” Studies were classified as low (0–5 points), medium (6–7 points), or high quality (8–11 points).

### 2.5 Statistical analysis

STATA 18.0 was used for data analysis and forest plots were plotted to visually display the pooled results. We preferentially extracted data from each study with adjusted OR values and 95%CI to assess the risk of cognitive impairment in patients with overt hypothyroidism. Heterogeneity was assessed with the use of the chi-square test and I2 values, with p values of less than 0.1 or I2 values of more than 50% considered to indicate heterogeneity, with the use of a random-effects model (28). Otherwise, a fixed effects model was used (29). Subgroup analyses were performed on the basis of population characteristics, study types, criteria for overt hypothyroidism, and whether adjustment for vascular disease to account for potential heterogeneity. In addition, sensitivity analyses were performed to verify the robustness of the overall results. Finally, funnel plots and Egger’s test were used to detect publication bias (30).

## 3 Results

### 3.1 Literature search

**Figure 1:**
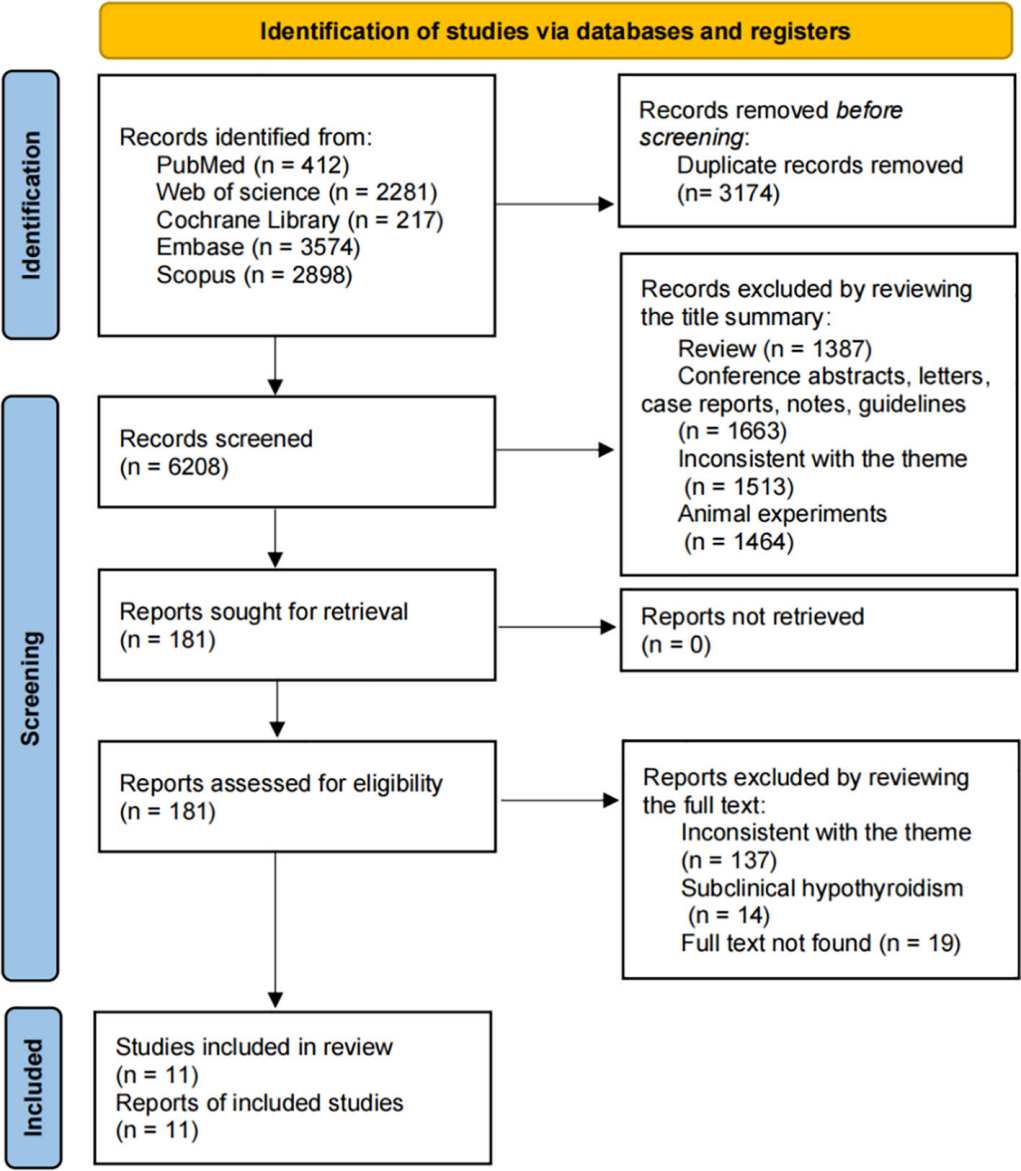
Flow diagram of literature selection for the meta-analysis.

The search process is summarized in **Figure 1**. A total of 9,382 articles were identified, with 6,208 remaining after duplicate removal. After title and abstract screening, 181 articles were considered potentially relevant. Following full-text review, 11 studies from 9 articles were included in the meta-analysis (14, 17–19, 31–35).

### 3.2 Study characteristics

The characteristics of the included studies are presented in **Table 1**. The 11 studies [2 of which included 2 cohorts (33, 35)] comprised 4 case-control studies, 4 cohort studies, and 3 cross-sectional studies. These studies involved diverse populations from China, Korea, Finland, Denmark, the United States, and Mexico, published between 2010 and 2022, and included 1,190,059 participants. The age range of participants was 33.5 to 85 years, with females comprising 44.2% to 82% of the samples. Follow-up durations in cohort studies ranged from 6.2 to 21.9 years. The primary outcome was MCI in 4 studies and dementia in 7 studies (3 all-cause dementia, 3 AD, and 1 either all-cause dementia or AD). Only one study did not adjust for potential confounders; common adjustment variables included age, gender, education, BMI, smoking, hypertension, and diabetes. In addition, the quality assessments of the individual studies are provided in **Supplementary Tables S2–S4**.

**Table 1:** Characteristics of studies included in the meta-analysis.

Study	Type	Country	Sample Size	Female (%)	Age (years)^a^	Follow-up Years	Diagnosis of hypothyroidism	Diagnosis of cognitive disorder	Type of cognitive disorder	Adjusted factors
Hu et al. (MCI), (35)	Case-control	China	141(77:64)	46.8%:45.3%	64.1(10.4):64.3(9.6)	NA	TSH>4.78mIU/l, FT4<11.50pmol/l, FT3<3.50pmol/l	Peterson standard	MCI	Age, sex, years of education, and BMI
Hu et al. (AD), (35)	Case-control	China	231(77:154)	46.8%:44.2%	64.1(10.4):63.5(9.4)	NA	TSH>4.78mIU/l, FT4<11.50pmol/l, FT3<3.50pmol/l	CDR	AD	Age, sex, years of education, and BMI
Kim et al. (32)	Case-control	Korea	82365(65892:16473)	61.1%:61.1%	NA	NA	ICD-10 codes	ICD-10 codes	AD	Obesity, smoking, alcohol consumption, systolic blood pressure, diastolic blood pressure, fasting blood glucose, total cholesterol, hemoglobin, thyroid cancer, CCI score, levothyroxine treatment, goiter, hypothyroidism, thyroiditis, and hyperthyroidism
Wieland et al. (17)	Case-control	China	15686(7843:7843)	51.8%	74.5(11.3):74.9(11.3)	NA	ICD-9 codes	ICD-9 codes	Dementia	Gender, age, history of hypertension, diabetes mellitus, coronary heart disease, depression, hyperlipidemic arterial disease, hyperlipidemia, alcohol dependence syndrome, tinnitus, hearing loss, and radioiodine treatment
Sipilä et al. (34)	Cohort Study	Finland	283414	69.1%	33.5 (18.0–87.9)	19	ICD-10 codes	ICD-10 codes	Dementia	sex and modifiable risk factors (low education/socioeconomic status, hypertension, obesity, smoking, depression, physical inactivity, marital status, and diabetes) and significant interactions between the adjusting variables
Thvilum et al. (DNPR), (33)	Cohort Study	Denmark	557825	82%	Hypothyroid: 55.8 (43.3–68.5) Reference: 55.8 (43.3–68.5)	6.2	At least two consecutive elevated TSH values (> 4.0 mIU/L) within 6 months with at least 14 days between measurements	ICD-10 codes	AD, Dementia	CCI score
Thvilum et al. (OPENTHYRO), (33)	Cohort Study	Denmark	233844	56%	Hypothyroid: 56.4 (42.6–69.2) References 50.3 (36.4–64.2)	7.2	ICD-10 codes	ICD-10 codes	Dementia	NA
George et al. (18)	Cohort Study	USA	12481	56.1%	Hypothyroidism:58.4(5.4) Reference: 56.8(5.7)	21.9	TSH>5.1mIU/L; FT4<0.85ng/dL	MMSE, CDR, FAQ, telephone interviews, ICD-9 codes, death-certificate codes	Dementia	Age, sex, race-center, APOE ϵ4, income and education, BMI, smoking status, hypertension, diabetes, drinking status, HDL-C and total cholesterol, prevalent CVD, and baseline thyroid medication use
Parsaik et al. (19)	Cross-section	USA	1904	48.6%%	Hypothyroidism: 81.20 (76.69–85.01) Reference: 80.03 (75.10–83.81)	NA	ICD-9 codes or HICDA code or TSH level≥10mIU/L and free thyroxine level<1.01 ng/dL	CDR and FAQ	MCI	age at visit, years of education, any APOE ϵ4 allele, Beck Depression Inventory depression, diabetes, hypertension, stroke, body mass index, coronary artery disease, and smoking
Juárez et al. (14)	Cross-section	Mexico	1750	60.6%	Hypothyroidism:73.3 (8.3) Reference: 72.1 (7.9)	NA	TSH>4.8 IU/l, FT4<13 pmol/l	MMSE	MCI	Age, sex, and years of education
Kim et al. (31)	Cross-section	Korea	495	61%	72.4 (5.6)	NA	TSH> 4.50 mIU/L	CSID	MCI	Age, sex, and education, smoking and physical activity, systolic blood pressure, diabetes, total cholesterol, and albumin, levothyroxine treatment, and depression

AD, Alzheimer’s Disease; MCI, mild cognitive impairment; TSH, thyroid-stimulating hormone; FT3, Free Triiodothyronine; FT4, Free Thyroxine; ICD, International Classification of Diseases; HICDA, International Classification of Diseases, Eighth Revision, Adapted Codes for Hospitals; MMSE, Mini-Mental Status Examination; CDR, Clinical Dementia Rating; FAQ, Functional Activities Questionnaire; CSID, Community Screening Interview for Dementia; BMI, body mass index; CCI, Charlson Comorbidity Index; CVD, cardiovascular disease; APOE, apolipoprotein E; HDL-C, high density lipoprotein cholesterol; NA, not available.

Data on Sample size, Female (%), and Age (years) of the case-control study were presented as healthy group: cognitive impairment group.

a Data are presented as mean (SD), median (range), and median (IQR) if available.

### 3.3 oHT and risk of CI

**Figure 2:**
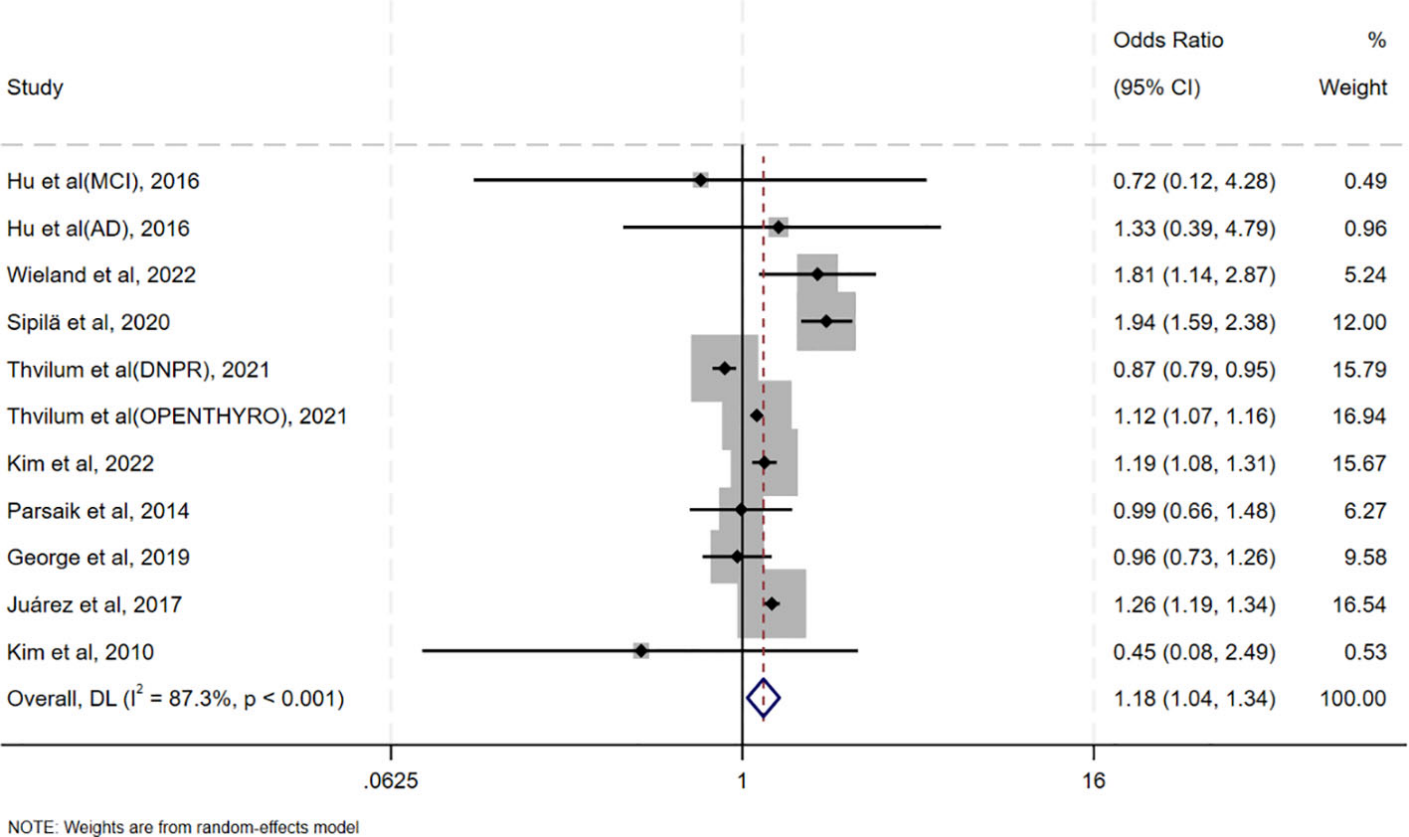
Forest plot of the association between oHT and CI.

Pooled analysis of ORs adjusted for the maximum number of covariates showed that oHT was associated with an increased risk of CI (OR = 1.18, 95%CI=1.04–1.34, I²=87.3%, p<0.001; **Figure 2**). It is noteworthy that the meta-analysis reveals a high degree of statistical heterogeneity (I² =87.3%, p <0.001), indicating significant differences in the effect sizes among the included studies, rather than due to sampling error. Therefore, interpretation of the pooled effect size should be approached with caution, and there is an urgent need for an in-depth exploration of the sources of heterogeneity. Sensitivity analysis in **Supplementary Figure S1** revealed that excluding individual studies resulted in point estimates ranging from 0.96 to 1.38, with 95% CIs consistently crossing 1, indicating non-significant results. However, point estimates slightly above 1 suggest a potential positive effect. A pooled analysis of unadjusted ORs yielded similar results (OR = 1.23, 95% CI = 1.12–1.35, I²=75.9%, p<0.001; **Supplementary Figure S2**).

### 3.4 oHT and risk of MCI, AD and dementia

**Figure 3:**
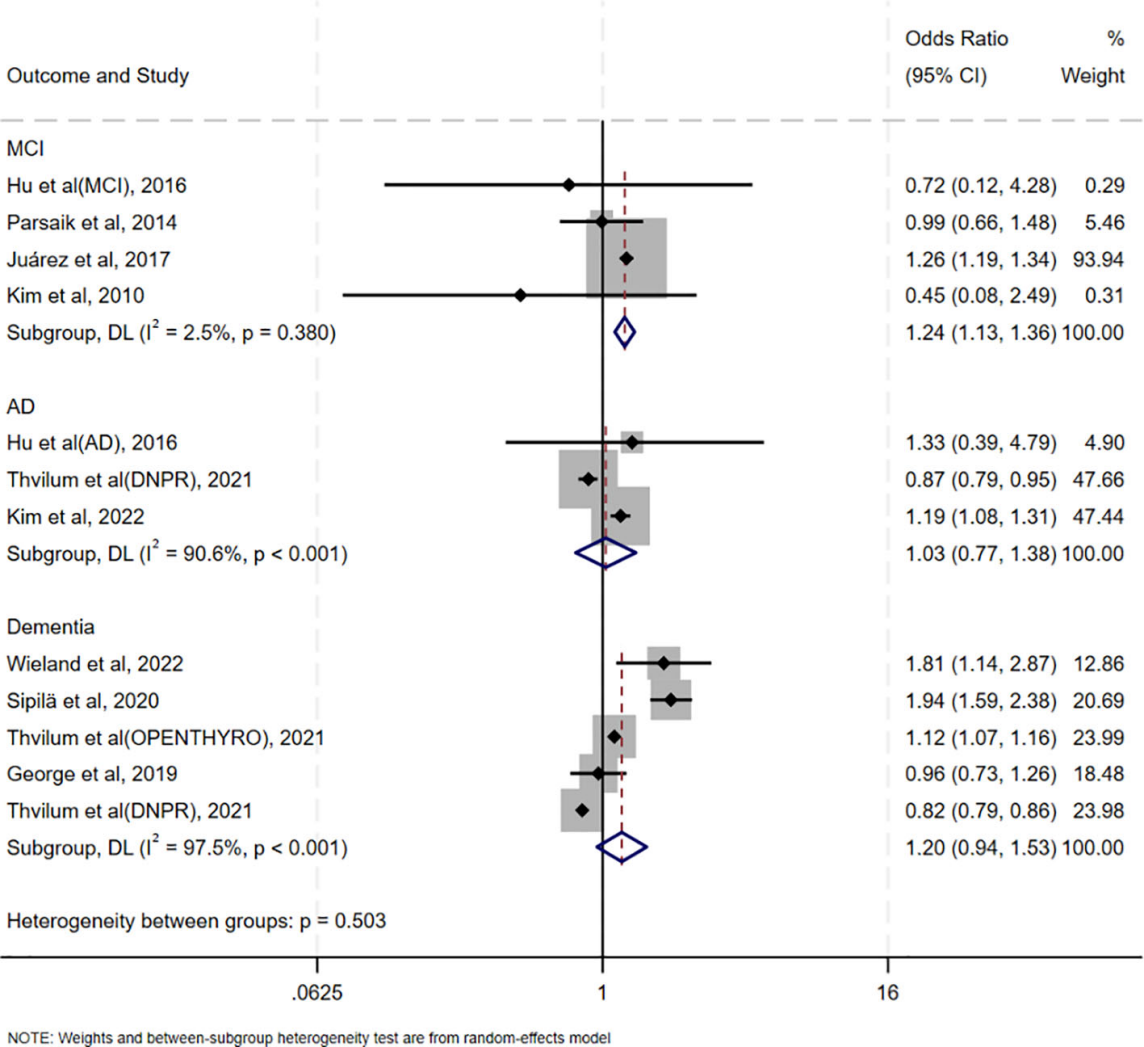
Forest plot of the association between oHT and different degrees of CI outcomes.

The association between oHT and MCI was evaluated in four studies conducted in China, the United States, Mexico, and Korea, respectively. Notably, these studies used diverse cognitive assessment tools (Peterson criteria, CDR, MMSE, and CSID), yet showed consistent direction of effect, supporting the robustness of the association. (OR = 1.24, 95% CI = 1.13–1.36, I² = 2.5%, p = 0.380; **Figure 3**). Three studies assessed the association between oHT and AD risk, revealing no significant increase (OR = 1.03, 95%CI=0.77–1.38, I²=90.6%, p<0.001; **Figure 3**). Five studies examined the association between oHT and all-cause dementia risk, also showing no significant increase (OR = 1.20, 95%CI=0.94–1.53, I²=97.5%, p<0.001; **Figure 3**).

### 3.5 Subgroup analysis

**Figure 4:**
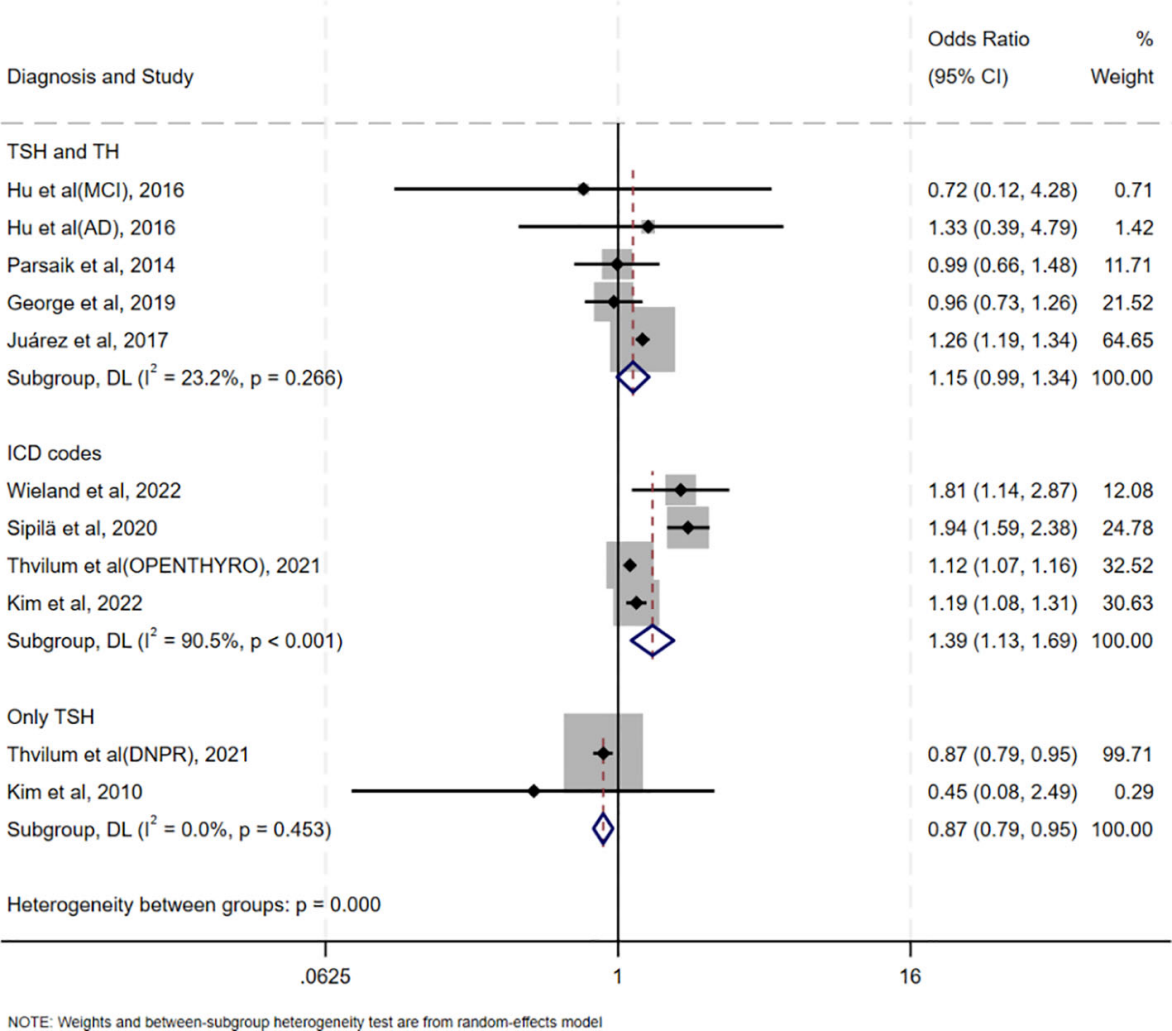
Forest plots of subgroup analyses according to different diagnostic criteria for oHT.

**Figure 5:**
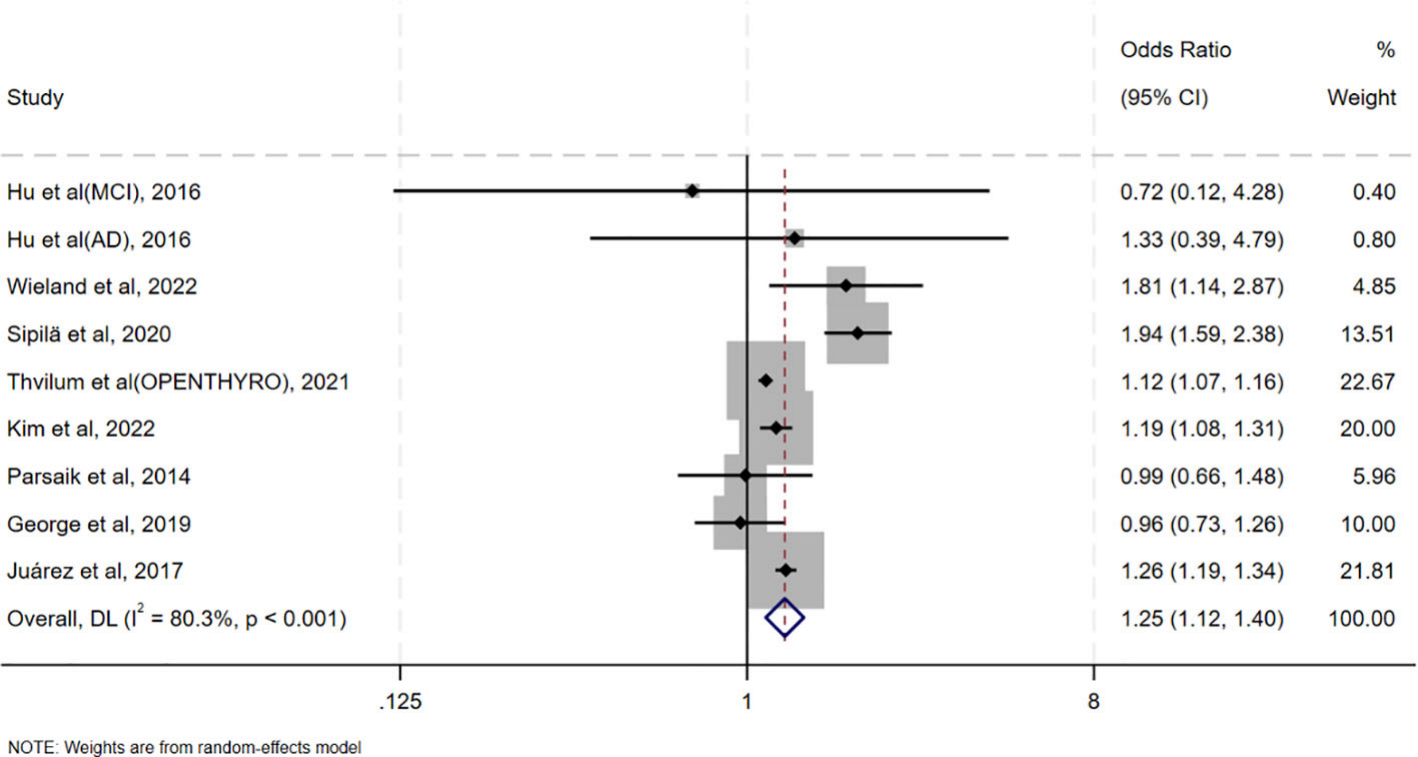
Forest plots to reexamine the association between oHT and CI after excluding studies reporting only TSH.

**Table 2** summarizes the results of all subgroup analyses to explore potential sources of heterogeneity, including population characteristics (region, age distribution, proportion of females, sample size), study type, diagnostic methods for oHT, and adjustment factors. Significant between-group differences were observed only in diagnostic methods for oHT (p<0.001; **Figure 4**). Specifically, studies using thyroid stimulating hormone (TSH) and thyroid hormone (TH) levels for diagnosis showed a pooled effect size of 1.15 (95% CI = 0.99–1.34, I² = 23.2%), while those using ICD codes had a pooled effect size of 1.39 (95% CI = 1.13–1.69, I² = 90.5%). Studies using only TSH levels for diagnosis showed a reduced risk of CI (OR = 0.87, 95% CI = 0.79–0.95, I² = 0.0%). Excluding these studies yielded a pooled effect size of 1.25 (95%CI=1.12–1.40, I² = 80.3%, p < 0.001; **Figure 5**). The gradient change in effect size caused by different diagnostic methods (ICD code>TSH+TH>TSH only) strongly indicates that differences in diagnostic methodology are the main reason for the appearance of “inconsistent” overall results.

**Table 2:** Subgroup analysis for the risk of CI in patients with oHT.

Subgroups		Numbers	OR(95%CI)	I^2^(%)	p values	p values between groups
Diagnostic methods for oHT	TSH and TH	5	1.15(0.99,1.34)	23.2%	0.266	<0.001
	ICD codes	4	1.39(1.13,1.69)	90.5%	<0.001	
	TSH	2	0.87(0.79,0.95)	0.0%	0.453	
Type of study	Case-control	4	1.26(1.04,1.53)	11.3%	0.336	0.823
	Cohort	4	1.15(0.91,1.47)	94.7%	<0.001	
	Cross-section	3	1.18(0.95,1.46)	26.2%	0.258	
Region	Asia	5	1.26(1.01,1.58)	14.0%	0.325	0.732
	Northern Europe	3	1.21(0.91,1.61)	96.4%	<0.001	
	North America	3	1.12(0.91,1.37)	58.7%	0.089	
Age (years)	<65	6	1.15(0.91,1.45)	91.3%	<0.001	0.612
	≥65	4	1.25(0.99,1.60)	41.3%	0.164	
Female (%)	<60%	6	1.12(0.99,1.26)	17.2%	0.302	0.497
	≥60%	5	1.22(0.97,1.54)	94.3%	<0.001	
Sample size	<10000	5	1.25(1.18,1.33)	0.0%	0.543	0.667
	≥10000	6	1.20(1.01,1.43)	92.2%	<0.001	
Adjustment of vascular diseases	yes	5	1.07(0.92,1.24)	88.4%	<0.001	0.188
	no	6	1.35(0.99,1.84)	84.8%	<0.001	

In other subgroup analyses, case-control studies showed that oHT was associated with an increased risk of CI (OR = 1.26, 95%CI=1.04–1.53, I²=11.3%, p=0.336; **Supplementary Figure S3**), whereas cohort and cross-sectional studies did not. oHT was associated with an increased risk of CI in Asian populations (OR = 1.26, 95%CI=1.01–1.58, I²=14.0%, p=0.325; **Supplementary Figure S4**) but not in Northern European or North American populations. Subgroup analyses based on age or proportion of females did not show a significant association between oHT and CI risk (**Supplementary Figures S5, S6**). Both small (OR = 1.25, 95%CI=1.18–1.33, I²=0.0%, p=0.543; **Supplementary Figure S7**) and large sample sizes (OR = 1.20, 95%CI=1.01–1.43, I²=92.2%, p<0.001; **Supplementary Figure S7**) suggested an increased risk of CI. Additionally, cerebrovascular disease, a significant risk factor for CI, was adjusted for in some studies, but oHT was not associated with an increased risk of CI in these analyses (**Supplementary Figure S8**).

### 3.6 Publication bias

**Figure 6:**
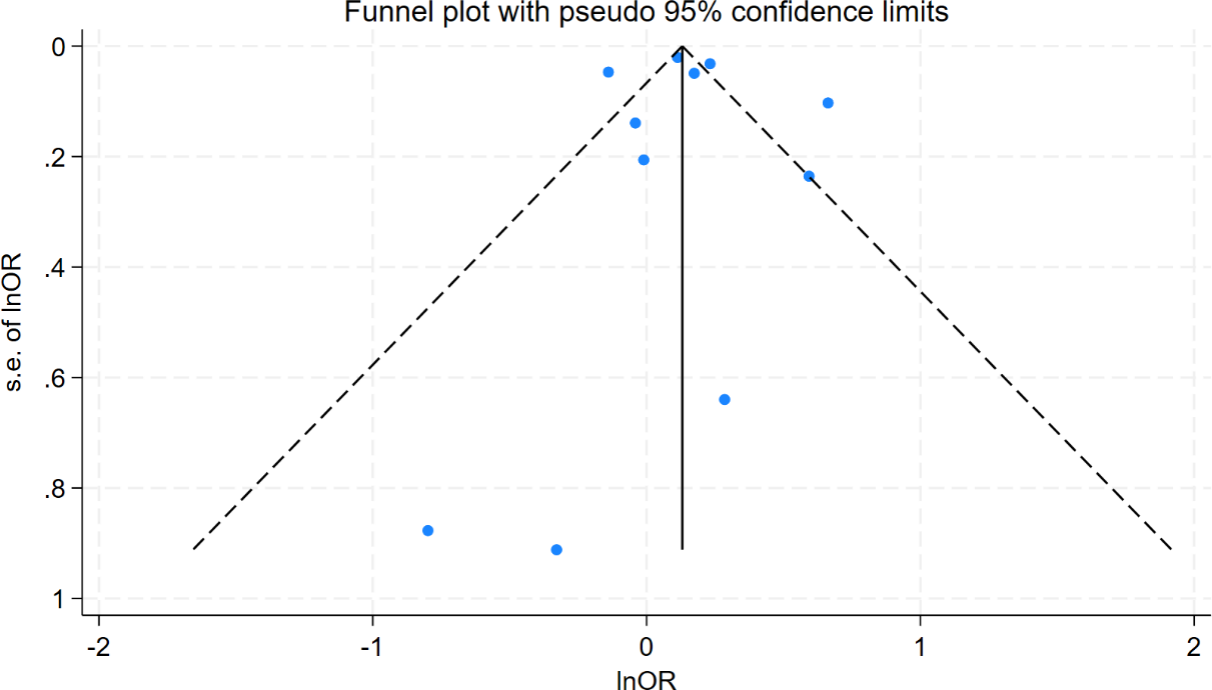
Funnel plot of standard errors by lnOR.

Funnel plot analysis showed symmetrical distribution of study points, with no significant outliers (**Figure 6**), suggesting low publication bias. Egger’s test further supported this finding (intercept coefficient=0.24, 95%CI=-2.53–3.01, p=0.849; **Table 3**), indicating no significant publication bias. Thus, the results of this meta-analysis are considered reliable.

**Table 3:** Egger’s test was used to assess publication bias.

Variable	Coefficient	Std. Err.	t	p-value	95% CI
Intercept	0.24	1.22	0.20	0.849	-2.53-3.01
Precision	0.12	0.06	2.00	0.077	-0.02-0.26

## 4 Discussion

This meta-analysis aimed to explore the relationship between oHT and CI risk. To our knowledge, this is the first meta-analysis specifically focusing on the association between oHT and CI. As a preliminary study, it provides an initial estimate of the association and, more importantly, highlights critical methodological sources of heterogeneity that must be addressed in future work. The pooled analysis of 11 studies demonstrated that oHT was associated with an increased risk of CI, whether using adjusted (OR = 1.18, 95%CI=1.04–1.34) or unadjusted OR values (OR = 1.23, 95% CI = 1.12–1.35). This suggests that oHT may be an independent risk factor for CI. Further analysis by CI severity revealed that oHT was associated with an increased risk of MCI (OR = 1.24, 95% CI = 1.13–1.36) but not with AD (OR = 1.03, 95%CI=0.77–1.38) or all-cause dementia (OR = 1.20, 95%CI=0.94–1.53). Sensitivity analysis revealed that excluding individual studies resulted in point estimates ranging from 0.96 to 1.38, with 95% CIs consistently crossing 1, indicating that the statistical significance of the pooled effect was not robust. However, most point estimates remained above 1.0, suggesting a potential positive trend.

From a biological perspective, thyroid hormones play a critical role in maintaining brain function (36–39). They regulate neuronal metabolism, synaptic plasticity, neurotransmitter synthesis and release, and neuronal survival and differentiation (40–42). In oHT patients, significantly reduced thyroid hormone levels may slow neuronal metabolism, impairing energy supply and functional maintenance. Additionally, thyroid hormone deficiency can decrease synaptic plasticity (43–45), disrupting neuronal signaling and further impairing cognitive functions such as memory, learning, and executive function. Beyond direct neuronal effects, oHT may contribute to CI through indirect pathways, including impaired cerebrovascular function, increased oxidative stress (46–48), and heightened inflammatory responses (49–51). These mechanisms support the association between oHT and CI and highlight the potential importance of identifying and managing oHT. However, given the observational nature and limitations of this study, our findings do not directly demonstrate that intervening in oHT can effectively prevent dementia. This critical issue urgently needs to be addressed through rigorously designed randomized controlled trials (RCTs).

Our findings align with some previous studies showing an increased risk of CI with oHT (52, 53), while others report no significant association (19). This inconsistency may stem from differences in study design, sample characteristics, and diagnostic criteria for oHT. For instance, some studies relied solely on TSH levels for oHT diagnosis, whereas our subgroup analysis found that studies using combined TSH and TH levels showed a more significant effect size (OR = 1.15, 95%CI=0.99–1.34) compared to those using only TSH (OR = 0.87, 95%CI=0.79–0.95). This highlights the importance of diagnostic criteria in influencing study outcomes. Previous meta-analyses often combined various forms of hypothyroidism, such as subclinical hypothyroidism, overt hypothyroidism, and thyroiditis, without rigorously distinguishing between overt and subclinical hypothyroidism. This approach likely introduced confounding factors that obscured the true relationship between overt hypothyroidism and cognitive impairment. Additionally, the limited number of studies focusing specifically on overt hypothyroidism in prior analyses may have further reduced the statistical power to detect a significant association, potentially contributing to the negative conclusions drawn. In contrast, the present study specifically focused on overt hypothyroidism and analyzed it independently, thereby providing a clearer and more precise understanding of its potential association with cognitive impairment.

The high heterogeneity found in this study (I²=87.3%) is a core issue that needs to be considered when interpreting the results. Our subgroup analysis successfully identified the diagnostic method of oHT as its main source. This difference may stem from the fundamentally different patient populations captured by different diagnostic methods. The study using ICD codes is likely to include oHT patients who seek medical attention due to clinical symptoms, have a longer course of illness, are more severe, or have more complications, therefore their assessed risk is the highest (OR = 1.39). On the contrary, studies that rely solely on a single TSH elevation diagnosis may inadvertently include some subclinical, temporary, or mild thyroid dysfunction patients, thereby diluting the true risk and even obtaining a reverse association (OR = 0.87). The study using TSH combined with THs levels for diagnosis has the most accurate patient definition, and its risk estimate (OR = 1.15) may be the closest to the true association. Although diagnostic methods explain most of the heterogeneity, residual heterogeneity may still be related to population characteristics (such as age, race), assessment tools for cognitive impairment, and differences in the degree of adjustment for confounding factors (such as vascular disease). Therefore, the summary results of this study should be considered as an average of the reported effect sizes under different clinical backgrounds and research methods, which reveals the existence of associations but also emphasizes that their strength may vary significantly in different contexts. Funnel plot and Egger’s test revealed no significant publication bias (Egger’s test p=0.849), indicating that the results of this meta-analysis are highly reliable. Nevertheless, attention should be paid to other potential sources of bias, such as differences in study design and inadequate adjustment for confounding factors. In particular, some studies did not adjust for important confounders such as vascular comorbidities, which may have affected the accuracy of the results.

Although this meta-analysis provides valuable evidence supporting the association between overt hypothyroidism (oHT) and cognitive impairment (CI), several limitations should be acknowledged. First, the high heterogeneity among the included studies may compromise the stability of the results. Second, the vast majority of the original studies we included did not provide more detailed clinical characteristic data of the oHT patient population. Especially, there is a lack of information regarding the specific etiology of oHT, the use of levothyroxine, and the adequacy of treatment. Untreated oHT, under treatment but poorly controlled oHT, and adequately treated oHT may have different potential impact mechanisms and risk intensities on cognitive function. Combining all these patients with different states for analysis may lead to underestimation or misestimation of the true risk, which is another potential important source of heterogeneity observed in this study. Third, the geographic distribution of the included studies was predominantly limited to Asia, Northern Europe, and North America, potentially restricting the generalizability of the results to other populations. Fourth, the small sample sizes in some studies may have reduced the precision of the effect size estimates. To address these limitations, future research should have the following characteristics to provide higher-level evidence: (a) systematically collect data on the etiology, treatment status, treatment dose, treatment duration, and thyroid function (TSH) levels during oHT follow-up; (b) Adopting a standardized oHT diagnostic process based on international guidelines (which must be based on both TSH and free thyroxine FT4 levels) to ensure the accuracy of patient grouping and avoid mistakenly including subclinical hypothyroidism patients; (c) Large scale, multi-center samples to provide sufficient statistical power to detect effect size and allow for sufficient subgroup analysis.

## 5 Conclusion

Available evidence suggests an association between oHT and an increased risk of cognitive impairment, particularly MCI. However, given the observational nature and significant heterogeneity of this study, the strength of this association still requires high-quality prospective studies for final confirmation and precise quantification.

## Data Availability

The original contributions presented in the study are included in the article/**Supplementary Material**. Further inquiries can be directed to the corresponding author.
