# Supplementary material for: Association of overt hypothyroidism with risks of cognitive impairment: a meta-analysis and systematic review
Source: Front Endocrinol (Lausanne). 2025 Oct 1;16:1643589. doi: 10.3389/fendo.2025.1643589 (PMC12520926; doi:10.3389/fendo.2025.1643589)
Supplement: Supplementary file 1 [file Supplementaryfile1.docx]

**Association of overt hypothyroidism with risks of cognitive impairment: a meta-analysis and systematic review**

**Supplementary Table 1. The complete literature search strategy**

1. **Pubmed**

| **Steps** | **Content** | **Results** |
| --- | --- | --- |
| #1 | ((((((((((((Hypothyroidism[MeSH Terms]) OR (Hypothyroidism[Title/Abstract])) OR (Hypothyroidisms[Title/Abstract])) OR (Thyroid-Stimulating Hormone Deficiency[Title/Abstract])) OR (Thyroid-Stimulating Hormone Deficiencies[Title/Abstract])) OR (TSH Deficiency[Title/Abstract])) OR (TSH Deficiencies[Title/Abstract])) OR (Primary Hypothyroidism[Title/Abstract])) OR (Primary Hypothyroidisms[Title/Abstract])) OR (Thyroid Dysfunction[Title/Abstract])) OR (Thyroid Dysfunctions[Title/Abstract])) OR (Subclinical Hypothyroidism[Title/Abstract])) OR (Subclinical Hypothyroidisms[Title/Abstract]) | 57757 |
| #2 | ((((((((((((Cognitive Dysfunction[MeSH Terms]) OR (Cognitive Dysfunction[Title/Abstract])) OR (Cognitive Dysfunctions[Title/Abstract])) OR (Cognitive Disorder[Title/Abstract])) OR (Cognitive Disorders[Title/Abstract])) OR (Cognitive Impairment[Title/Abstract])) OR (Cognitive Impairments[Title/Abstract])) OR (Mild Cognitive Impairment[Title/Abstract])) OR (Mild Cognitive Impairments[Title/Abstract])) OR (Cognitive Decline[Title/Abstract])) OR (Cognitive Declines[Title/Abstract])) OR (Mental Deterioration[Title/Abstract])) OR (Mental Deteriorations[Title/Abstract]) | 164202 |
| #3 | #1 AND #2 | 412 |

1. **Web of science**

| **Steps** | **Content** | **Results** |
| --- | --- | --- |
| #1 | (((((((((((TS=(Hypothyroidism)) OR TS=(Hypothyroidisms)) OR TS=(Thyroid-Stimulating Hormone Deficiency)) OR TS=(Thyroid-Stimulating Hormone Deficiencies)) OR TS=(TSH Deficiency)) OR TS=(TSH Deficiencies)) OR TS=(Primary Hypothyroidism)) OR TS=(Primary Hypothyroidisms)) OR TS=(Thyroid Dysfunction)) OR TS=(Thyroid Dysfunctions)) OR TS=(Subclinical Hypothyroidism)) OR TS=(Subclinical Hypothyroidisms) and Preprint Citation Index (Exclude – Database) | 90493 |
| #2 | (((((((((((TS=(Cognitive Dysfunction)) OR TS=(Cognitive Dysfunctions)) OR TS=(Cognitive Disorder)) OR TS=(Cognitive Disorders)) OR TS=(Cognitive Impairments)) OR TS=(Cognitive Impairment)) OR TS=(Mild Cognitive Impairment)) OR TS=(Mild Cognitive Impairments)) OR TS=(Cognitive Decline)) OR TS=(Cognitive Declines)) OR TS=(Mental Deterioration)) OR TS=(Mental Deteriorations) and Preprint Citation Index (Exclude – Database) | 706489 |
| #3 | #1 AND #2 and Preprint Citation Index (Exclude – Database) | 2281 |

1. **Scopus**

| **Steps** | **Content** | **Results** |
| --- | --- | --- |
| #1 | ( TITLE-ABS-KEY ( hypothyroidism ) OR TITLE-ABS-KEY ( hypothyroidisms ) OR TITLE-ABS-KEY ( &apos;thyroid-stimulating AND hormone AND deficiencies&apos; ) OR TITLE-ABS-KEY ( &apos;thyroid-stimulating AND hormone AND deficiency&apos; ) OR TITLE-ABS-KEY ( &apos;tsh AND deficiencies&apos; ) OR TITLE-ABS-KEY ( &apos;tsh AND deficiency&apos; ) OR TITLE-ABS-KEY ( &apos;primary AND hypothyroidisms&apos; ) OR TITLE-ABS-KEY ( &apos;primary AND hypothyroidism&apos; ) OR TITLE-ABS-KEY ( &apos;thyroid AND dysfunction&apos; ) OR TITLE-ABS-KEY ( &apos;thyroid AND dysfunctions&apos; ) OR TITLE-ABS-KEY ( &apos;subclinical AND hypothyroidisms&apos; ) OR TITLE-ABS-KEY ( &apos;subclinical AND hypothyroidism&apos; ) ) | 105191 |
| #2 | ( TITLE-ABS-KEY ( &apos;cognitive AND dysfunction&apos; ) OR TITLE-ABS-KEY ( &apos;cognitive AND dysfunctions&apos; ) OR TITLE-ABS-KEY ( &apos;cognitive AND disorders&apos; ) OR TITLE-ABS-KEY ( &apos;cognitive AND disorder&apos; ) OR TITLE-ABS-KEY ( &apos;cognitive AND impairments&apos; ) OR TITLE-ABS-KEY ( &apos;cognitive AND impairment&apos; ) OR TITLE-ABS-KEY ( &apos;mild AND cognitive AND impairments&apos; ) OR TITLE-ABS-KEY ( &apos;mild AND cognitive AND impairment&apos; ) OR TITLE-ABS-KEY ( &apos;cognitive AND declines&apos; ) OR TITLE-ABS-KEY ( &apos;cognitive AND decline&apos; ) OR TITLE-ABS-KEY ( &apos;mental AND deteriorations&apos; ) OR TITLE-ABS-KEY ( &apos;mental AND deterioration&apos; ) ) | 483218 |
| #3 | #1 AND #2 | 2898 |

1. **Cochrane library**

| **Steps** | **Content** | **Results** |
| --- | --- | --- |
| #1 | MeSH descriptor: [Hypothyroidism] explode all trees | 636 |
| #2 | Hypothyroidisms | 3 |
| #3 | Thyroid-Stimulating Hormone Deficiency | 118 |
| #4 | Thyroid-Stimulating Hormone Deficiencies | 16 |
| #5 | TSH Deficiency | 302 |
| #6 | TSH Deficiencies | 25 |
| #7 | Primary Hypothyroidism | 1701 |
| #8 | Primary Hypothyroidisms | 2 |
| #9 | Thyroid Dysfunction | 906 |
| #10 | Thyroid Dysfunctions | 47 |
| #11 | Subclinical Hypothyroidism | 588 |
| #12 | Subclinical Hypothyroidisms | 1 |
| #13 | #1 OR #2 OR #3 OR #4 OR #5 OR #6 OR #7 OR #8 OR #9 OR #10 OR #11 OR #12 | 3300 |
| #14 | MeSH descriptor: [Cognitive Dysfunction] explode all trees | 4399 |
| #15 | Cognitive Dysfunctions | 610 |
| #16 | Cognitive Disorder | 26359 |
| #17 | Cognitive Disorders | 29560 |
| #18 | Cognitive Impairments | 4019 |
| #19 | Cognitive Impairment | 20188 |
| #20 | Mild Cognitive Impairment | 7402 |
| #21 | Mild Cognitive Impairments | 699 |
| #22 | Cognitive Decline | 7824 |
| #23 | Cognitive Declines | 759 |
| #24 | Mental Deterioration | 1905 |
| #25 | Mental Deteriorations | 31 |
| #26 | OR #15 OR #16 OR #17 OR #18 OR #19 OR #20 OR #21 OR #22 OR #23 OR #24 OR #25 | 61402 |
| #27 | #13 AND #26 | 217 |

1. **Embase**

| **Steps** | **Content** | **Results** |
| --- | --- | --- |
| #1 | 'hypothyroidism'/exp OR 'hypothyroidism' | 115561 |
| #2 | hypothyroidisms | 97 |
| #3 | 'thyroid stimulating' AND hormone AND deficiency | [2084](https://www-embase-com-443.webvpn.cams.tsgvip.top/) |
| #4 | 'thyroid stimulating' AND hormone AND deficiencies | [286](https://www-embase-com-443.webvpn.cams.tsgvip.top/) |
| #5 | tsh AND deficiency | [5801](https://www-embase-com-443.webvpn.cams.tsgvip.top/) |
| #6 | tsh AND deficiencies | [886](https://www-embase-com-443.webvpn.cams.tsgvip.top/) |
| #7 | primary AND hypothyroidism | [16,270](https://www-embase-com-443.webvpn.cams.tsgvip.top/) |
| #8 | primary AND hypothyroidisms | [21](https://www-embase-com-443.webvpn.cams.tsgvip.top/) |
| #9 | thyroid AND dysfunction | [22390](https://www-embase-com-443.webvpn.cams.tsgvip.top/) |
| #10 | thyroid AND dysfunctions | [1565](https://www-embase-com-443.webvpn.cams.tsgvip.top/) |
| #11 | subclinical AND hypothyroidism | [10890](https://www-embase-com-443.webvpn.cams.tsgvip.top/) |
| #12 | subclinical AND hypothyroidisms | [23](https://www-embase-com-443.webvpn.cams.tsgvip.top/) |
| #13 | #1 OR #2 OR #3 OR #4 OR #5 OR #6 OR #7 OR #8 OR #9 OR #10 OR #11 OR #12 | [132332](https://www-embase-com-443.webvpn.cams.tsgvip.top/) |
| #14 | cognitive AND dysfunction | [101739](https://www-embase-com-443.webvpn.cams.tsgvip.top/) |
| #15 | cognitive AND dysfunctions | [9442](https://www-embase-com-443.webvpn.cams.tsgvip.top/) |
| #16 | cognitive AND disorder | [248552](https://www-embase-com-443.webvpn.cams.tsgvip.top/) |
| #17 | cognitive AND disorders | [180928](https://www-embase-com-443.webvpn.cams.tsgvip.top/) |
| #18 | cognitive AND impairment | [219486](https://www-embase-com-443.webvpn.cams.tsgvip.top/) |
| #19 | cognitive AND impairments | [51279](https://www-embase-com-443.webvpn.cams.tsgvip.top/) |
| #20 | mild AND cognitive AND impairment | [66277](https://www-embase-com-443.webvpn.cams.tsgvip.top/) |
| #21 | mild AND cognitive AND impairments | [5777](https://www-embase-com-443.webvpn.cams.tsgvip.top/) |
| #22 | cognitive AND decline | [83558](https://www-embase-com-443.webvpn.cams.tsgvip.top/) |
| #23 | cognitive AND declines | [6602](https://www-embase-com-443.webvpn.cams.tsgvip.top/) |
| #24 | mental AND deterioration | [22224](https://www-embase-com-443.webvpn.cams.tsgvip.top/) |
| #25 | mental AND deteriorations | [323](https://www-embase-com-443.webvpn.cams.tsgvip.top/) |
| #26 | #14 OR #15 OR #16 OR #17 OR #18 OR #19 OR #20 OR #21 OR #22 OR #23 OR #24 OR #25 | [554694](https://www-embase-com-443.webvpn.cams.tsgvip.top/) |
| #27 | #13 AND #26 | [3574](https://www-embase-com-443.webvpn.cams.tsgvip.top/) |


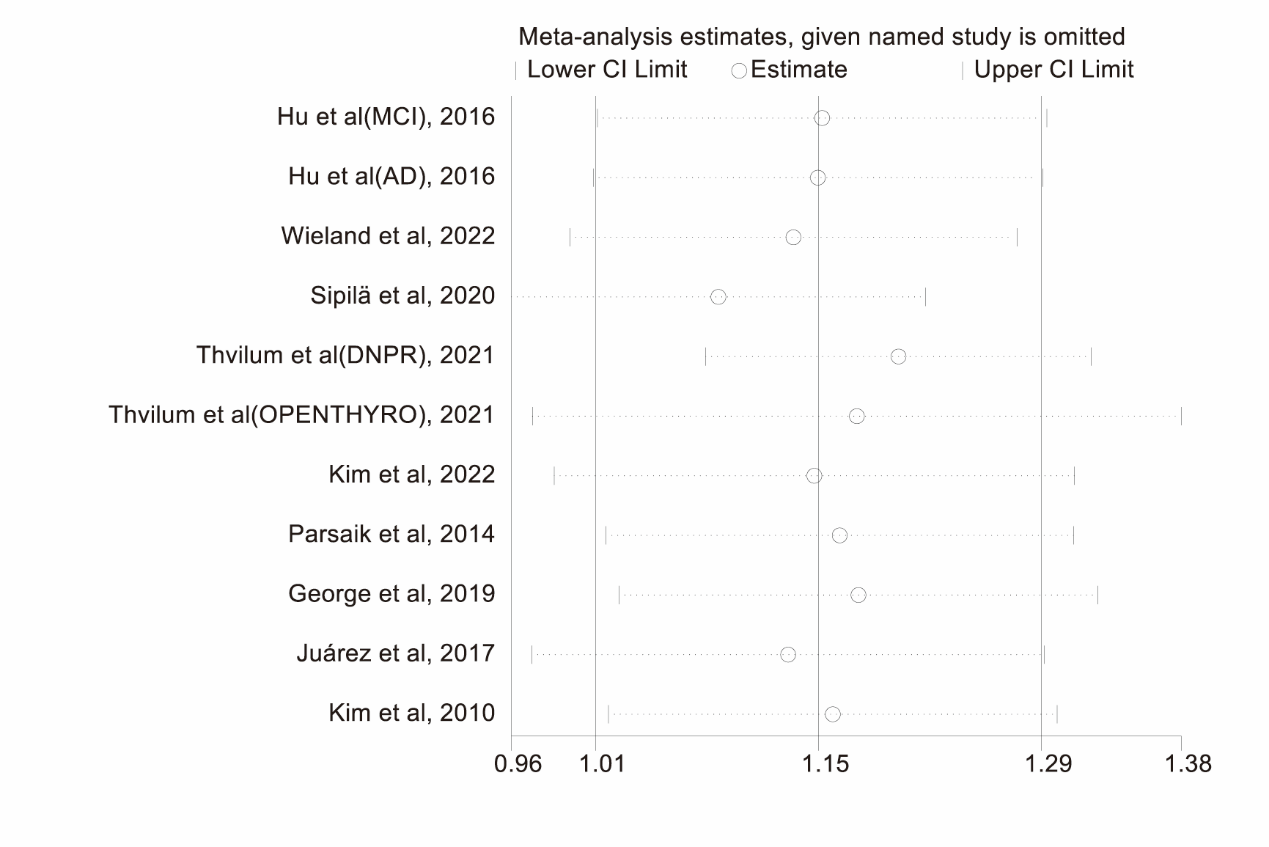


**Supplementary Figure 1. Sensitivity analysis of the risk of oHT and CI**


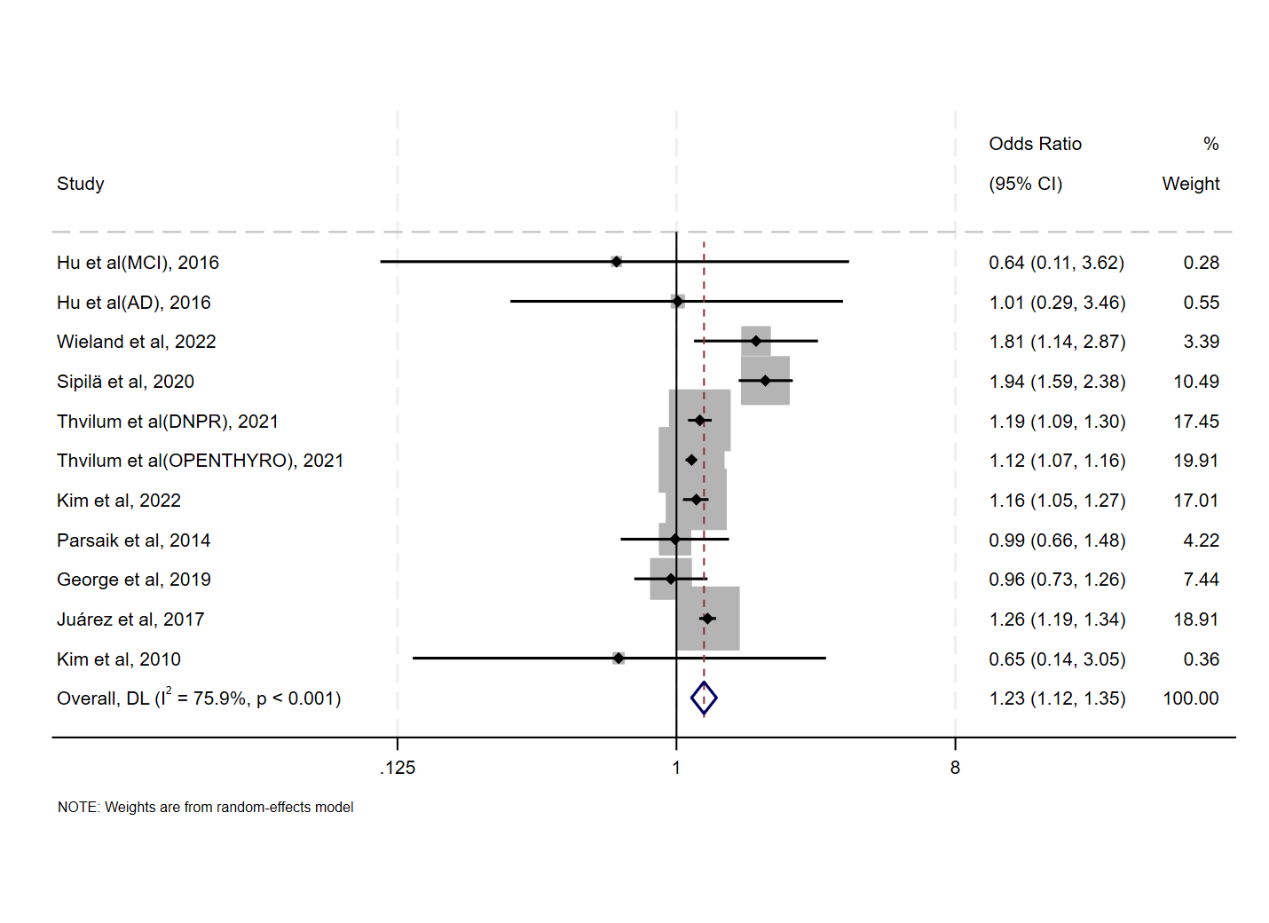


**Supplementary Figure 2. Forest plot of overt hypothyroidism and cognitive impairment(Unadjusted comorbidities were included).**


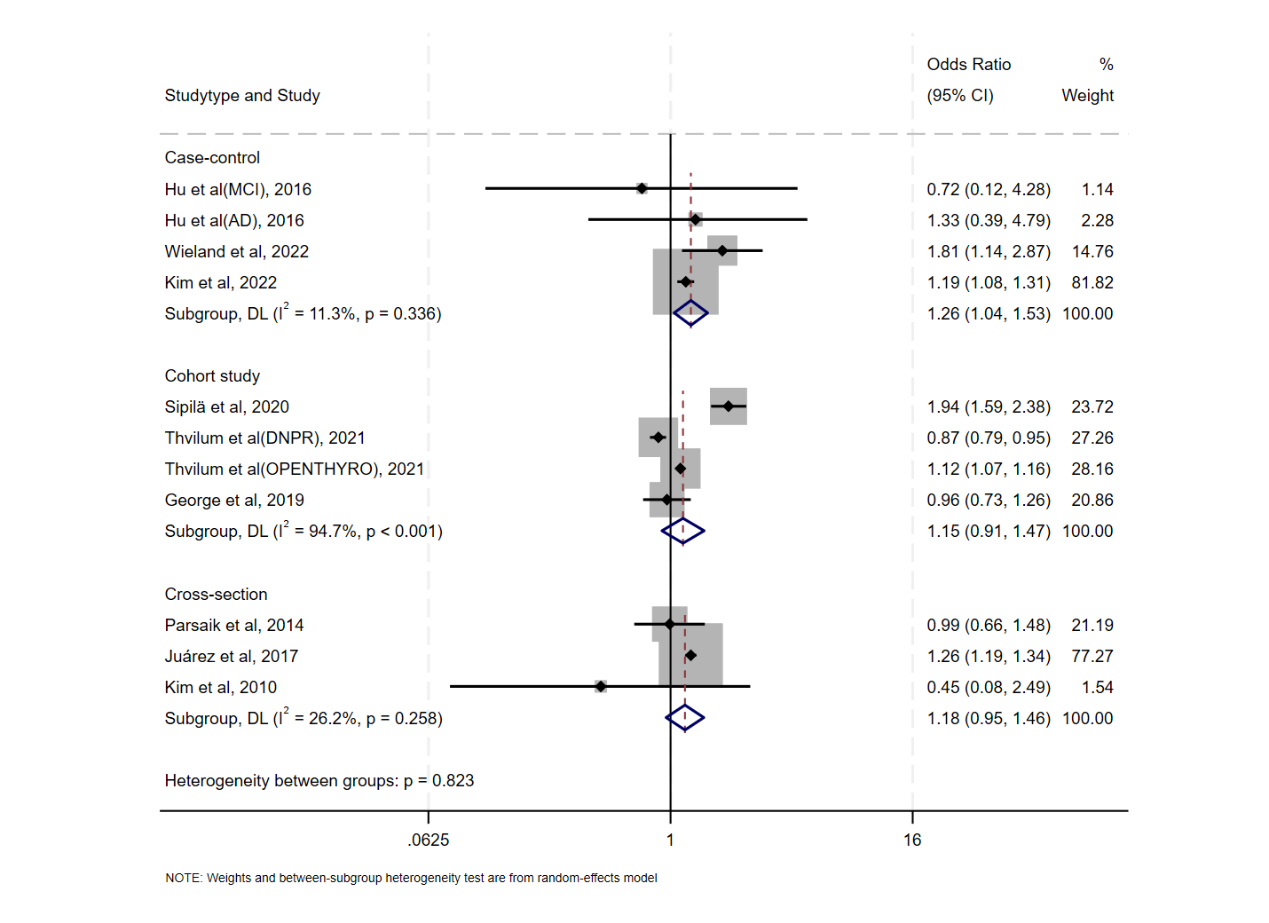


**Supplementary Figure 3. Forest plots of subgroup analyses according to different study types**


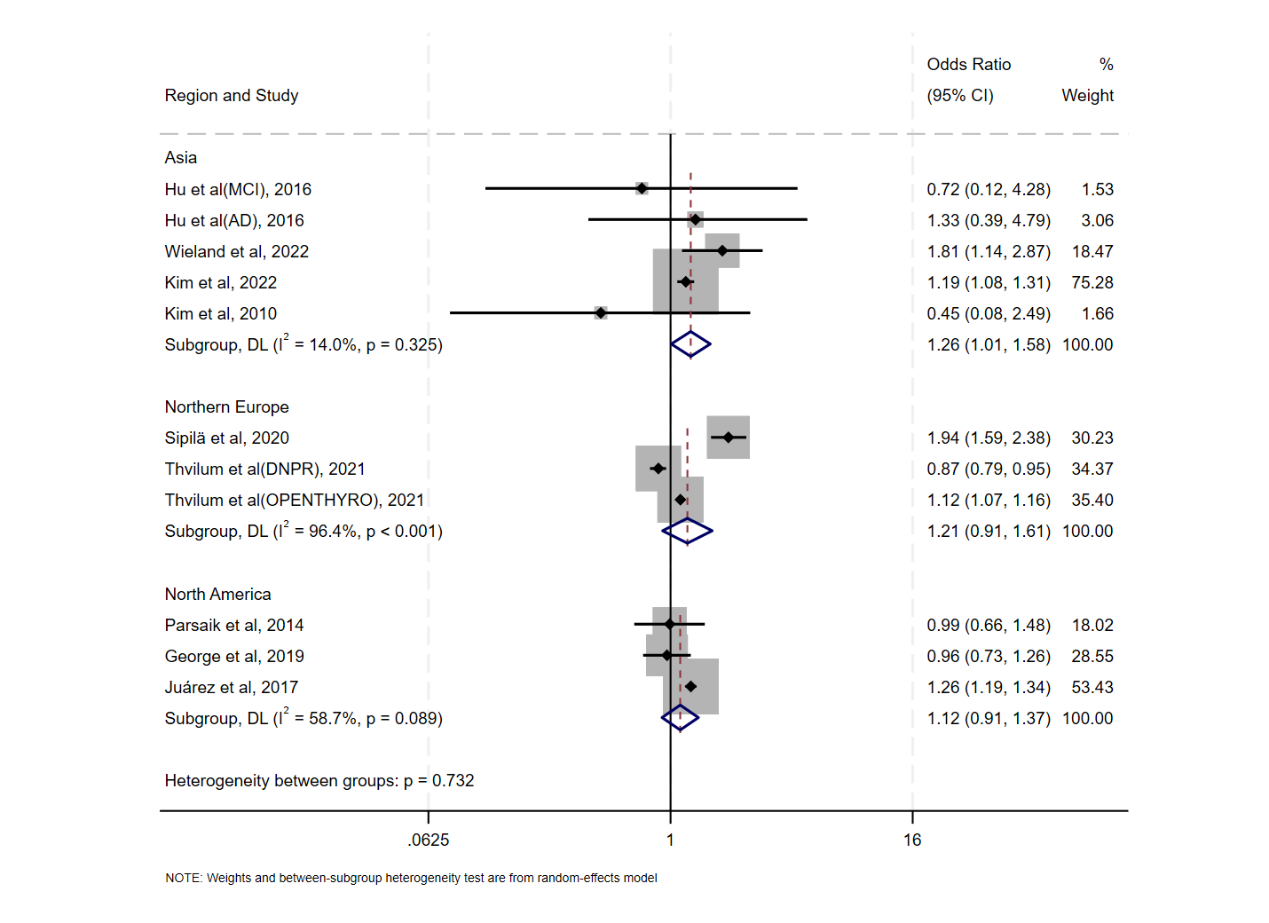


**Supplementary Figure 4. Forest plots for subgroup analysis according to the region of the population**


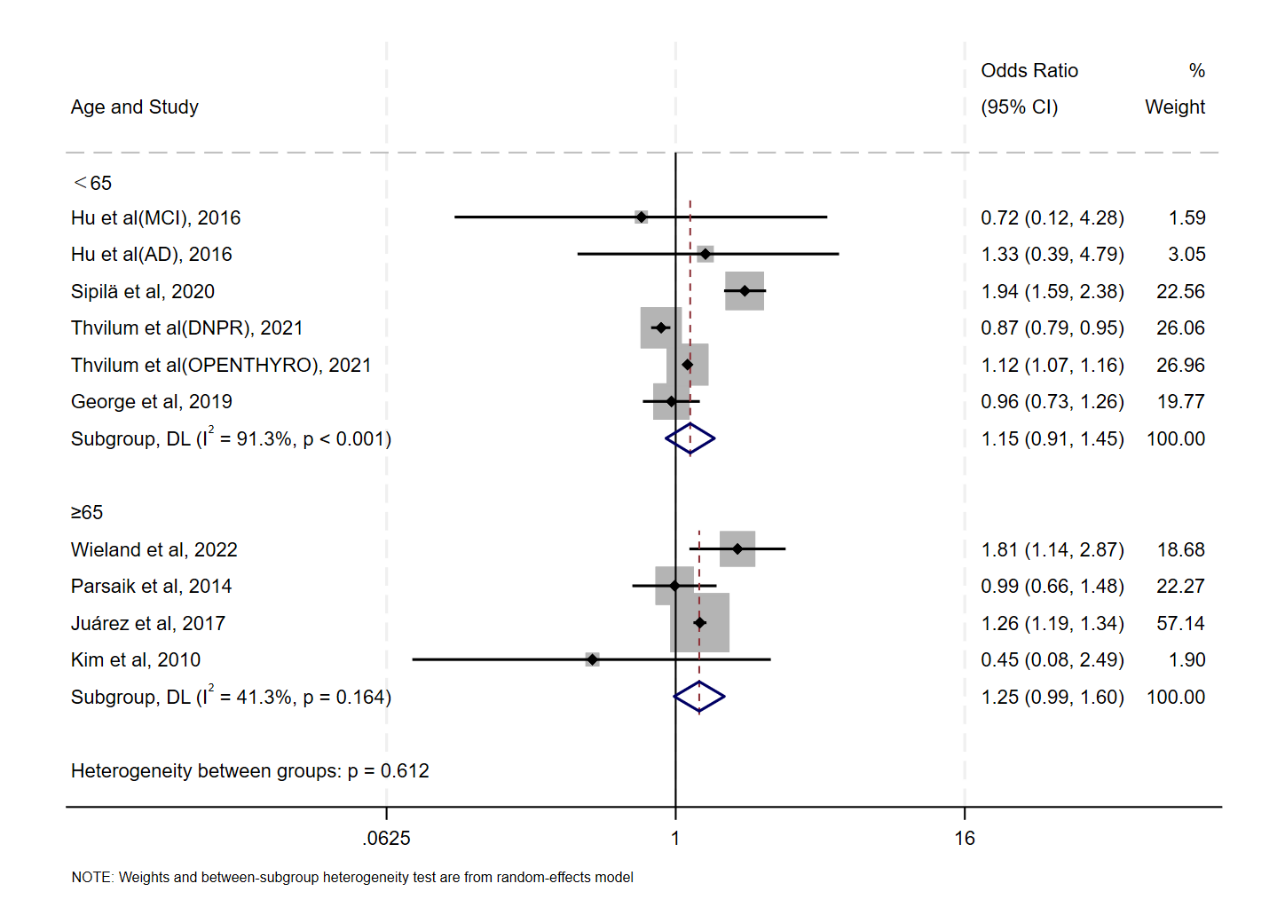


**Supplementary Figure 5. Forest plots of subgroup analyses according to population age distribution**


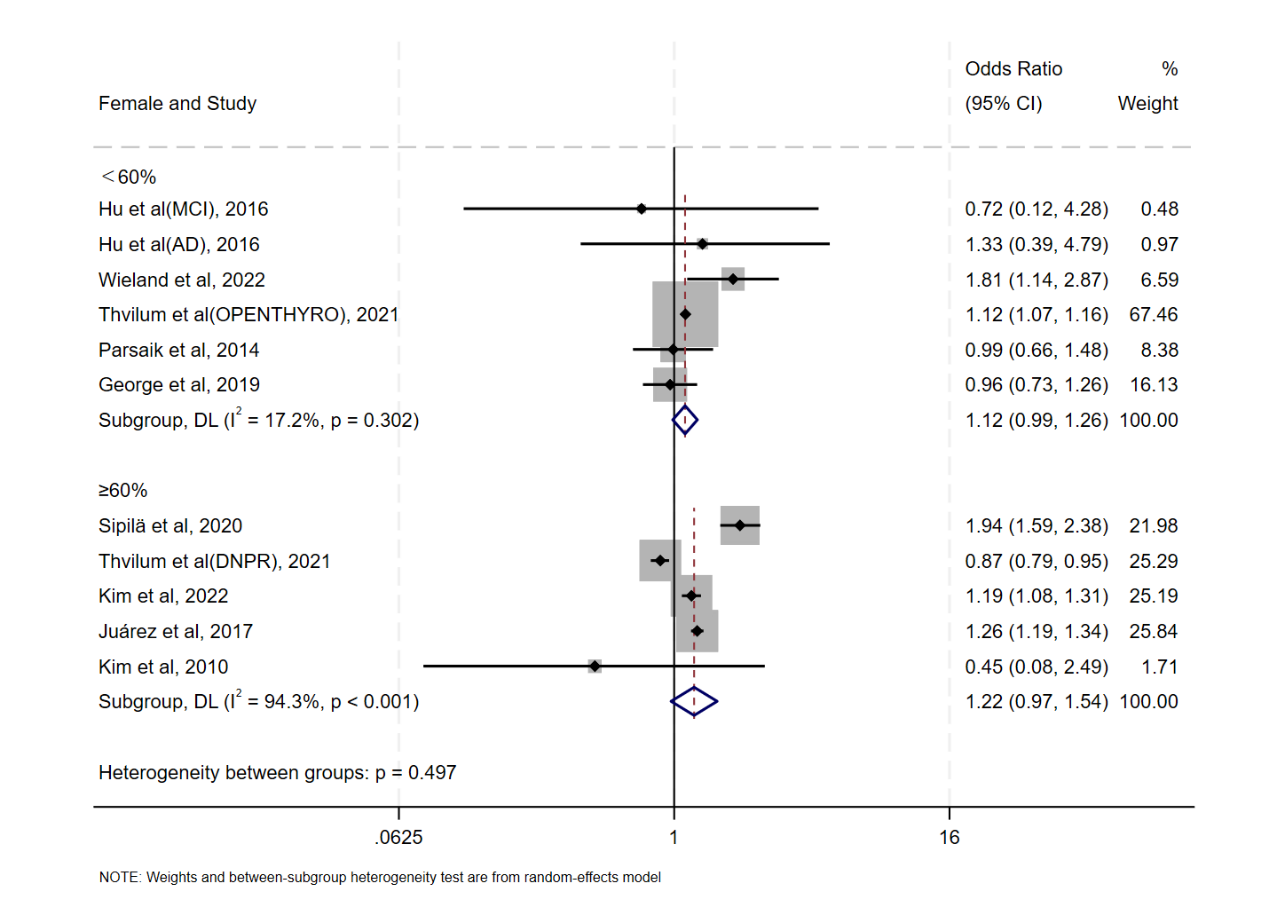


**Supplementary Figure 6. Forest plot of subgroup analysis according to the proportion of females in the population**


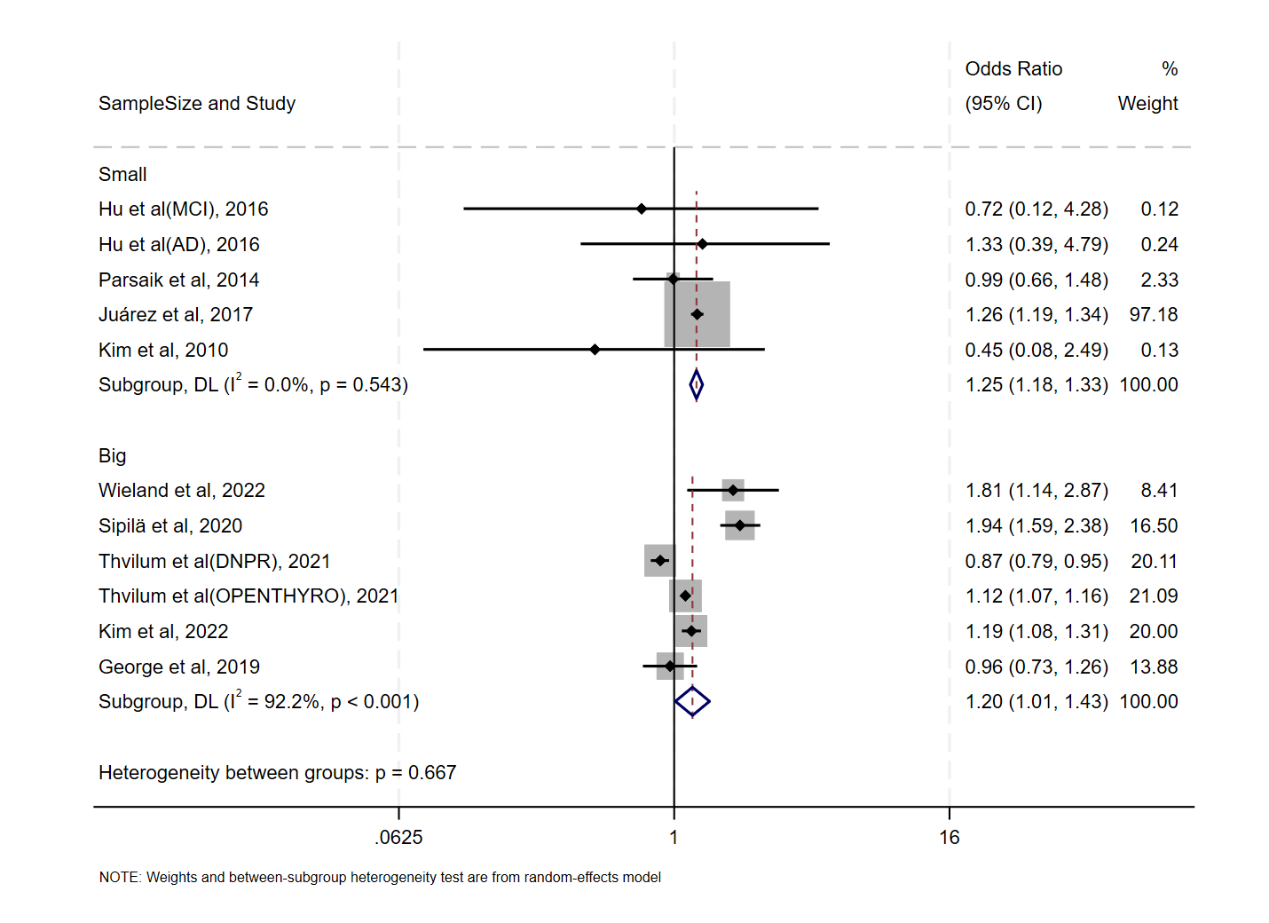


**Supplementary Figure 7. Forest plots of subgroup analyses according to sample size. Small sample: ≤ 10,000, large sample: > 10,000.**


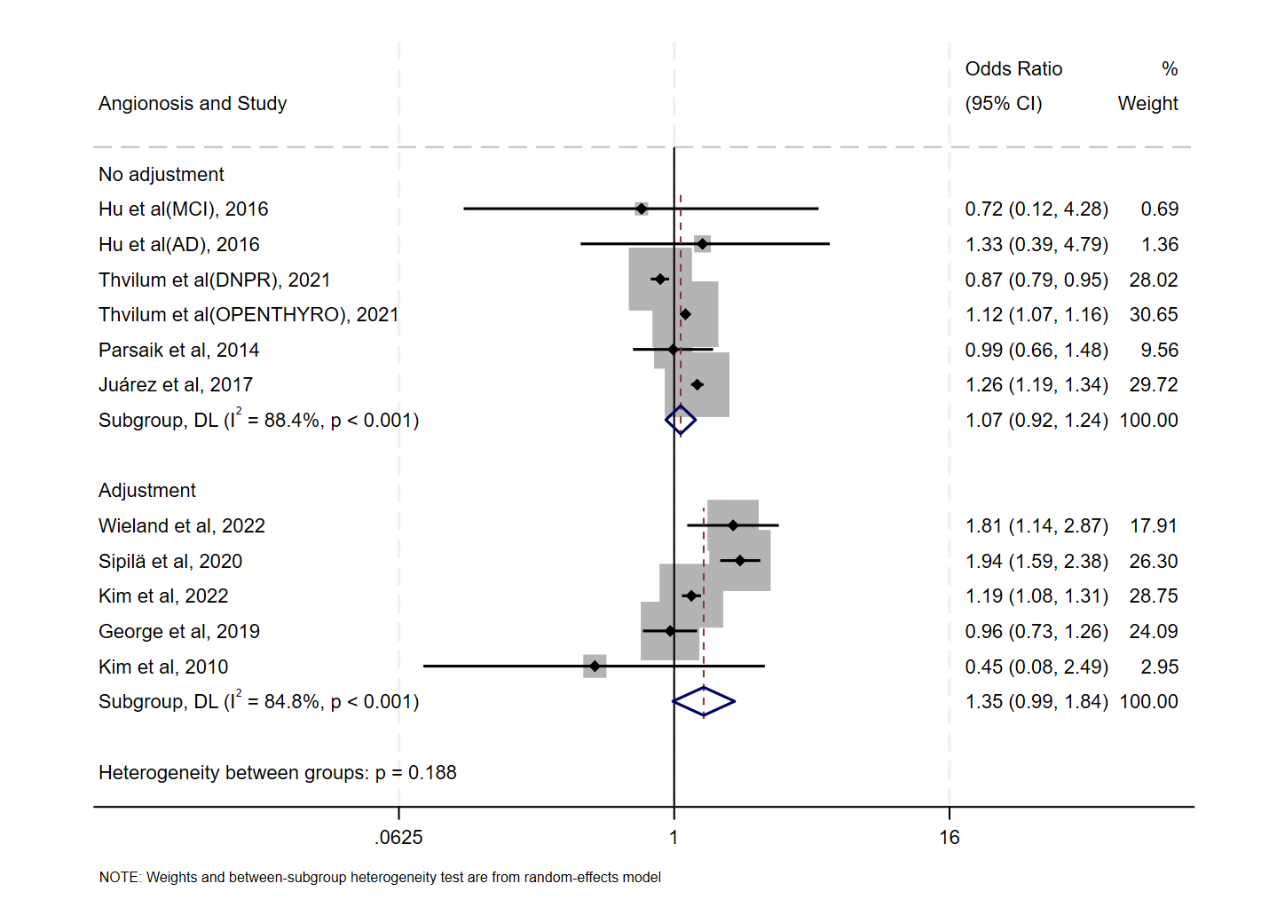


**Supplementary Figure 8. Forest plots of subgroup analyses according to whether adjustment was made for vascular comorbidities.**

**Supplementary Table 2. NOS scale scores for case-control studies**

| **Study** | **Selection** | **Comparability** | **Outcome** | **Overall quality score** |
| --- | --- | --- | --- | --- |
| Hu et al (MCI), 2016 | ★★★★ | ★ | ★★ | 7 |
| Hu et al (AD), 2016 | ★★★★ | ★ | ★★ | 7 |
| Kim et al, 2022 | ★★★★ | ★★ | ★★★ | 9 |
| Wieland et al, 2022 | ★★★★ | ★★ | ★★★ | 9 |

**Supplementary Table 3. NOS scale scores for cohort studies**

| **Study** | **Selection** | **Comparability** | **Outcome** | **Overall quality score** |
| --- | --- | --- | --- | --- |
| Sipilä et al, 2020 | ★★★★ | ★★ | ★★★ | 9 |
| Thvilum et al (DNPR), 2021 | ★★★★ | ★ | ★★ | 7 |
| Thvilum et al (OPENTHYRO), 2021 | ★★★★ | - | ★★★ | 7 |
| George et al, 2019 | ★★★★ | ★★ | ★★ | 8 |

**Supplementary Table 4. AHRQ scale scores for cross-sectional studies**

| **Study** | **Overall quality score** |
| --- | --- |
|  |  |
| Parsaik et al, 2014 | 9 |
| Juárez et al, 2017 | 9 |
| Kim et al, 2010 | 8 |

**Supplementary Table 5. Preferred Reporting Items for Systematic Reviews and Meta-Analyses (PRISMA) checklist.**

| **Section and Topic** | **Item #** | **Checklist item** | **Location where item is reported** |
| --- | --- | --- | --- |
| **TITLE** | | |  |
| Title | 1 | Identify the report as a systematic review. | Title |
| **ABSTRACT** | | |  |
| Abstract | 2 | See the PRISMA 2020 for Abstracts checklist. | Abstract |
| **INTRODUCTION** | | |  |
| Rationale | 3 | Describe the rationale for the review in the context of existing knowledge. | Paragraph 1-2 of the Introduction |
| Objectives | 4 | Provide an explicit statement of the objective(s) or question(s) the review addresses. | Paragraph 3 of the Introduction |
| **METHODS** | | |  |
| Eligibility criteria | 5 | Specify the inclusion and exclusion criteria for the review and how studies were grouped for the syntheses. | 2.3 Study Selection and Data Extraction |
| Information sources | 6 | Specify all databases, registers, websites, organisations, reference lists and other sources searched or consulted to identify studies. Specify the date when each source was last searched or consulted. | 2.2 Search Strategy |
| Search strategy | 7 | Present the full search strategies for all databases, registers and websites, including any filters and limits used. | 2.2 Search Strategy |
| Selection process | 8 | Specify the methods used to decide whether a study met the inclusion criteria of the review, including how many reviewers screened each record and each report retrieved, whether they worked independently, and if applicable, details of automation tools used in the process. | 2.3 Study Selection and Data Extraction Section, Paragraph 1 |
| Data collection process | 9 | Specify the methods used to collect data from reports, including how many reviewers collected data from each report, whether they worked independently, any processes for obtaining or confirming data from study investigators, and if applicable, details of automation tools used in the process. | 2.3 Study Selection and Data Extraction Section, Paragraph 1 |
| Data items | 10a | List and define all outcomes for which data were sought. Specify whether all results that were compatible with each outcome domain in each study were sought (e.g. for all measures, time points, analyses), and if not, the methods used to decide which results to collect. | 3.1 Literature Search and Table 1 |
|  | 10b | List and define all other variables for which data were sought (e.g. participant and intervention characteristics, funding sources). Describe any assumptions made about any missing or unclear information. | 3.1 Literature Search and Table 1 |
| Study risk of bias assessment | 11 | Specify the methods used to assess risk of bias in the included studies, including details of the tool(s) used, how many reviewers assessed each study and whether they worked independently, and if applicable, details of automation tools used in the process. | 2.5 Statistical analysis |
| Effect measures | 12 | Specify for each outcome the effect measure(s) (e.g. risk ratio, mean difference) used in the synthesis or presentation of results. | 2.5 Statistical analysis |
| Synthesis methods | 13a | Describe the processes used to decide which studies were eligible for each synthesis (e.g. tabulating the study intervention characteristics and comparing against the planned groups for each synthesis (item #5)). | 3.1 Literature Search and Figure 1 |
|  | 13b | Describe any methods required to prepare the data for presentation or synthesis, such as handling of missing summary statistics, or data conversions. | 2.5 Statistical analysis |
|  | 13c | Describe any methods used to tabulate or visually display results of individual studies and syntheses. | 2.5 Statistical analysis |
|  | 13d | Describe any methods used to synthesize results and provide a rationale for the choice(s). If meta-analysis was performed, describe the model(s), method(s) to identify the presence and extent of statistical heterogeneity, and software package(s) used. | 2.5 Statistical analysis |
|  | 13e | Describe any methods used to explore possible causes of heterogeneity among study results (e.g. subgroup analysis, meta-regression). | 2.5 Statistical analysis |
|  | 13f | Describe any sensitivity analyses conducted to assess robustness of the synthesized results. | 2.5 Statistical analysis |
| Reporting bias assessment | 14 | Describe any methods used to assess risk of bias due to missing results in a synthesis (arising from reporting biases). | 2.5 Statistical analysis |
| Certainty assessment | 15 | Describe any methods used to assess certainty (or confidence) in the body of evidence for an outcome. | 2.5 Statistical analysis |
| **RESULTS** | | |  |
| Study selection | 16a | Describe the results of the search and selection process, from the number of records identified in the search to the number of studies included in the review, ideally using a flow diagram. | 3.1 Literature Search and Figure 1 |
|  | 16b | Cite studies that might appear to meet the inclusion criteria, but which were excluded, and explain why they were excluded. | Figure 1 |
| Study characteristics | 17 | Cite each included study and present its characteristics. | 3.1 Literature Search and Table 1 |
| Risk of bias in studies | 18 | Present assessments of risk of bias for each included study. | 3.6 Publication Bias and Figure 6 |
| Results of individual studies | 19 | For all outcomes, present, for each study: (a) summary statistics for each group (where appropriate) and (b) an effect estimate and its precision (e.g. confidence/credible interval), ideally using structured tables or plots. | 3.3 oHT and Risk of CI 3.3 oHT and Risk of CI and Figure 2 |
| Results of syntheses | 20a | For each synthesis, briefly summarise the characteristics and risk of bias among contributing studies. | 3.2 Study Characteristics |
|  | 20b | Present results of all statistical syntheses conducted. If meta-analysis was done, present for each the summary estimate and its precision (e.g. confidence/credible interval) and measures of statistical heterogeneity. If comparing groups, describe the direction of the effect. | Sections 3.4 to 3.5 |
|  | 20c | Present results of all investigations of possible causes of heterogeneity among study results. | 3.2 Study Characteristics and Supplementary Figure 1 |
|  | 20d | Present results of all sensitivity analyses conducted to assess the robustness of the synthesized results. | Supplementary Figure 1 |
| Reporting biases | 21 | Present assessments of risk of bias due to missing results (arising from reporting biases) for each synthesis assessed. | 3.6 Publication Bias |
| Certainty of evidence | 22 | Present assessments of certainty (or confidence) in the body of evidence for each outcome assessed. | 95% CI were reported in all places involving OR |
| **DISCUSSION** | | |  |
| Discussion | 23a | Provide a general interpretation of the results in the context of other evidence. | The first paragraph of the discussion section |
|  | 23b | Discuss any limitations of the evidence included in the review. | Paragraph 5 of the discussion section |
|  | 23c | Discuss any limitations of the review processes used. | Paragraph 5 of the discussion section |
|  | 23d | Discuss implications of the results for practice, policy, and future research. | Paragraph 5 of the discussion section |
| **OTHER INFORMATION** | | |  |
| Registration and protocol | 24a | Provide registration information for the review, including register name and registration number, or state that the review was not registered. | 2.1 Standard Protocol Approvals, Registrations, and Guidelines |
|  | 24b | Indicate where the review protocol can be accessed, or state that a protocol was not prepared. | 2.1 Standard Protocol Approvals, Registrations, and Guidelines |
|  | 24c | Describe and explain any amendments to information provided at registration or in the protocol. | 2.1 Standard Protocol Approvals, Registrations, and Guidelines |
| Support | 25 | Describe sources of financial or non-financial support for the review, and the role of the funders or sponsors in the review. | Funding |
| Competing interests | 26 | Declare any competing interests of review authors. | Conflict of Interest |
| Availability of data, code and other materials | 27 | Report which of the following are publicly available and where they can be found: template data collection forms; data extracted from included studies; data used for all analyses; analytic code; any other materials used in the review. | Data Availability Statement |

**Supplementary Table 6. Subgroup analysis variables and their classification.**

| **Category variable name** | **Classification method** | **Definition basis** |
| --- | --- | --- |
| Diagnostic methods for oHT | TSH+THs, ICD code, TSH alone | Laboratory standards and medical records |
| Type of study | case control, cohort, cross section | research design |
| Region | Asia, northern Europe, North America | geographical distribution |
| Age (years) | <65 years old, ≥65 years old | Whether to enter the old age |
| Female (%) | <60%, ≥60% | Gender distribution differences |
| Sample size | <10,000, ≥10,000 | Statistical effectiveness |
| Adjustment of vascular diseases | yes, no | Whether to adjust hypertension, diabetes and stroke |
